# Identification of a Multicomponent Traditional Herbal Medicine by HPLC–MS and Electron and Light Microscopy

**DOI:** 10.3390/molecules22122242

**Published:** 2017-12-15

**Authors:** Ju-Han Liu, Yung-Yi Cheng, Chen-Hsi Hsieh, Tung-Hu Tsai

**Affiliations:** 1 Institute of Traditional Medicine, School of Medicine, National Yang-Ming University, Taipei 112, Taiwan; hanaa77721@gmail.com (J.-H.L.); vininecheng@gmail.com (Y.-Y.C.); chenciab@gmail.com (C.-H.H.); 2 Graduate Institute of Acupuncture Science, China Medical University, Taichung 404, Taiwan; 3 Division of Radiation Oncology, Department of Radiology, Far Eastern Memorial Hospital, Taipei 220, Taiwan; 4 Faculty of Medicine, School of Medicine, National Yang-Ming University, Taipei 112, Taiwan; 5 School of Pharmacy, College of Pharmacy, Kaohsiung Medical University, Kaohsiung 807, Taiwan; 6 Department of Chemical Engineering, National United University, Miaoli 36063, Taiwan

**Keywords:** herbal medicines, LC–MS, Xiang-Sha-Liu-Jun-Zi-Tang, SEM, Congo red stained, iodine–KI stained

## Abstract

Background: Commercial pharmaceutical herbal products have enabled people to take traditional Chinese medicine (TCM) in a convenient and accessible form. However, the quantity and quality should be additionally inspected. To address the issue, a combination of chemical and physical inspection methods were developed to evaluate the amount of an herbal formula, Xiang-Sha-Liu-Jun-Zi-Tang (XSLJZT), in clinical TCM practice. Methods: A high-performance liquid chromatography–tandem mass spectrometry (HPLC–MS) method with electrospray ionization was developed to measure the herbal biomarkers of guanosine, atractylenolide III, glycyrrhizic acid, dehydrocostus lactone, hesperidin, and oleanolic acid from XSLJZT. Scanning electron microscopy (SEM) photographs and light microscopy photographs with Congo red and iodine–KI staining were used to identify the cellulose fibers and starch content. Furthermore, solubility analysis, swelling power test, and crude fiber analysis were contributed to measure the starch additive in pharmaceutical products. Results: The results demonstrated large variations in the chemical components of different pharmaceutical brands. The SEM photographs revealed that the starch was oval, smooth, and granular, and that the raw herbal powder appears stripy, stretched, and filiform. The stained light microscopy photographs of all of the pharmaceutical products showed added starch and raw herbal powder as extenders. Conclusion: The developed chemical and physical methods provide a standard operating procedure for the quantity control of the herbal pharmaceutical products of XSLJZT.

## 1. Introduction

The technology of liquid chromatography–tandem mass spectrometry (LC–MS) has both qualitative and quantitative uses for classifying the chemical components in complex chemical mixtures [1]. These uses include identifying constituents and determining the structure of a compound by analyzing its fragmentation [2]. Additionally, MS ion fragments provide abundant structural information. In recent years, LC–MS has been widely used in traditional Chinese medicine (TCM) research because of its high selectivity, high sensitivity, and ability to generate specific information, including cognate molecular mass and structural characteristics [3]. TCM medicinal preparations generally contain a single herb or a mixture of two or more different types of medicinal herbs [4]. In addition, the complex ingredients of TCM could promote their clinical effects and security; therefore, separation and analysis of those chemical compositions are vital for the modernization of TCM [5]. Consequently, effective and specific methods for identifying multiple constituents of TCM have been developed.

For over a thousand years, TCM has held a critical role in treating various conditions in Asia. The worldwide population and the cost of traditional Chinese medicine therapy have grown dramatically in recent years [6,7]. Herbal products are composed of complex mixtures of organic materials that may come from some raw or processed parts of a plant, including the leaves, stems, flowers, roots, and seeds [8]. Nevertheless, quality controls for these herbal products still rarely demonstrate batch-to-batch production consistency, even for products from the same industrial pharmaceutical company [9]. In the Qing Dynasty, the classic TCM book *TCM Prescriptions*, written by well-known ancient and modern physicians, originally described Xiang-Sha-Liu-Jun-Zi-Tang (XSLJZT), which includes *Amomum villosum* and fresh costus roots (*Saussurea lappa*), as having the function of promoting Qi circulation through the composition of Liu-Jun-Zi-Tang (LJZT) [10]. XSLJZT is a phenomenally effective formula for cases involving spleen and stomach Qi deficiencies, with damp-cold stagnation inspiring the middle-jiao (middle burner). Symptoms and signs may include poor appetite, anorexia, general indigestion, abdominal distension or pain, vomiting or diarrhea, bloating after eating, and nausea [11]. XSLJZT can significantly stimulate gastrointestinal motility and gastric emptying, improve the electrogastrogram, adjust gastrointestinal hormones, and reduce gastric sensitivity [12]. According to Xiao’s research, XSLJZT is appropriate for the treatment of functional dyspepsia, due to the potential effects of multitarget therapy [13].

The National Health Insurance Research Database (NHIRD) in Taiwan has surveyed the herbal formulation of XSLJZT, which was based mostly on the Chinese herbal formula used for colon cancer patients post-surgery [14]. The herbal formulation XSLJZT consists of *Radix Aucklandiae* (Chinese herbal name: Muxiang), *Fructus Amomi* (Chinese herbal name: Sha-Ren), *Rhizoma Pinelliae* (Chinese herbal name: Ban-Xia), *Pericarpium Citri Reticulatae* (Chinese herbal name: Chenpi), *Radix Ginseng* (Chinese herbal name: Ren-Shen), *Atractylodes Rhizoma* (Chinese herbal name: Baizhu), *Poria cocos* (Schw.) *Wolf* (Chinese herbal name: Fu-Ling), *Glycyrrhizae Radix* (Chinese herbal name: Gan-Cai), and *Ziziphus jujuba* (Chinese herbal name: Da-Zao). The herbal formulation is derived from *Summarizing Songs on Popular Formulas* by Xiu-yuan Chen, which is a classical TCM herbal formulation [15]. *Radix Aucklandiae* specifically resolves stagnant Qi of the spleen, stomach, and intestines, which alleviates abdominal pain and discomfort [16]. *Fructus Amomi* is commonly used to treat morning sickness, but it is particularly effective for reducing nausea.

Recent investigations confirmed the effects of Sha-Ren for relieving abdominal spasms, bloating, and pain [17,18]. *Rhizoma Pinelliae* has antiemetic, glandular, antitussive, secretion-inhibiting, and antitumor effects, according to modern pharmacological studies [19]. *Pericarpium Citri Reticulatae* has a potent antineuroinflammatory ability that is attributed to the collective effects of nobiletin, hesperidin and tangeretin [20]. In addition, research has indicated that hesperidin has a protective effect against cisplatin-induced renal injury in rats [21]. Ginsenosides are found to be almost exclusively in the plant *Radix Ginseng*, which has a long history of use in traditional medicine. Many studies discovered that ginsenosides have antioxidant properties, such as increasing internal antioxidant enzymes and acting as a free-radical scavenger [22]. *Atractylodes Rhizoma* is the rootstock of *Atractylodes lancea* (Thunb.) DC. or *Atractylodes chinensis* (DC.) Koidz., and they are part of the *Asteraceae* family. It has gastroprotective and neuroprotective activities as well as antibacterial, antiviral, anti-inflammatory and anti-allergic properties [23]. The herb *Poria cocos* (Schw.) *Wolf* (*Polyporaceae*) grows around the roots of pine trees, and it is a famous traditional East Asian medicinal plant in China, Japan, Korea, and North America [24]. *Poria cocos Wolf* is used in the treatment of acute gastroenteric catarrh, chronic gastritis, edema, nephrosis, nausea, emesis, and dizziness [25]. With a wide spectrum of antiviral activity, glycyrrhizic acid (GA) is considered to be the principal component in *Glycyrrhiza* spp., and the roots have been used for the treatment of viral-induced hepatitis, cough, and skin diseases for thousands of years. [26]. *Ziziphus jujuba* is an important plant in popular medicine, and has been shown to present beneficial nutritional and health-promoting effects [27]. The major biologically active compounds include flavonoids, triterpene acids, polysaccharides, vitamin C, and phenolics [28]. Jujube fruits have been discovered by recent phytochemical studies to have biological effects, such as anti-inflammatory, antioxidant, anti-cancer, hepatoprotective, and gastrointestinal protective activities [28,29,30].

The popularity of TCM is increasing, but preparing TCMs is a time-consuming process. Therefore, herbal manufacturers produce instant herbal products to offer to consumers using TCM. In order to standardize high-quality herbal merchandise, quantitatively measuring the ingredients of TCM products with validated means is a long-term program in Taiwan. Under current regulations, it has been legal to add herbal powder to herbal pharmaceutical products to increase the contents of herbal ingredients [9]. As a result, the plants’ raw powder, starch, and cellulose are deliberately intermixed with herbal extract for granulation and volume extension. On the other hand, the simple milled raw herbal powder can contain pesticide residues, heavy metals, and bacterial contamination. Those concerning problems have been regulated by the manufacturers themselves. Therefore, measurement of the major active biomarker contents and herbal additives in Chinese herbal pharmaceutical products is necessary for assessing the quantity [31].

The aim of this investigation is to examine the quality of various brands of pharmaceutical products with scanning electron microscopy; light microscopy with Congo red and potassium iodide staining; a solubility test; and crude fiber analysis to examine the particle appearance, solubility, and the contents of crude fibers in order to identify the herbal formulation. To address these issues, we describe a high-performance liquid chromatography–tandem mass spectrometry (HPLC-MS) assay with multiple reaction monitoring (MRM) for the measurement of major biomarkers of guanosine, atractylenolide III, glycyrrhizic acid, dehydrocostus lactone, hesperidin, and oleanolic acid for the chemical examination of XSLJZT. The tested Chinese herbal pharmaceutical products of XSLJZT are made by six manufacturers. The systemic chemical and physical methods that were developed provide a standard procedure for conducting quality control of Chinese herbal pharmaceutical products.

## 2. Results and Discussion

### 2.1. Optimization of LC-MS/MS Conditions

**Figure 1:**
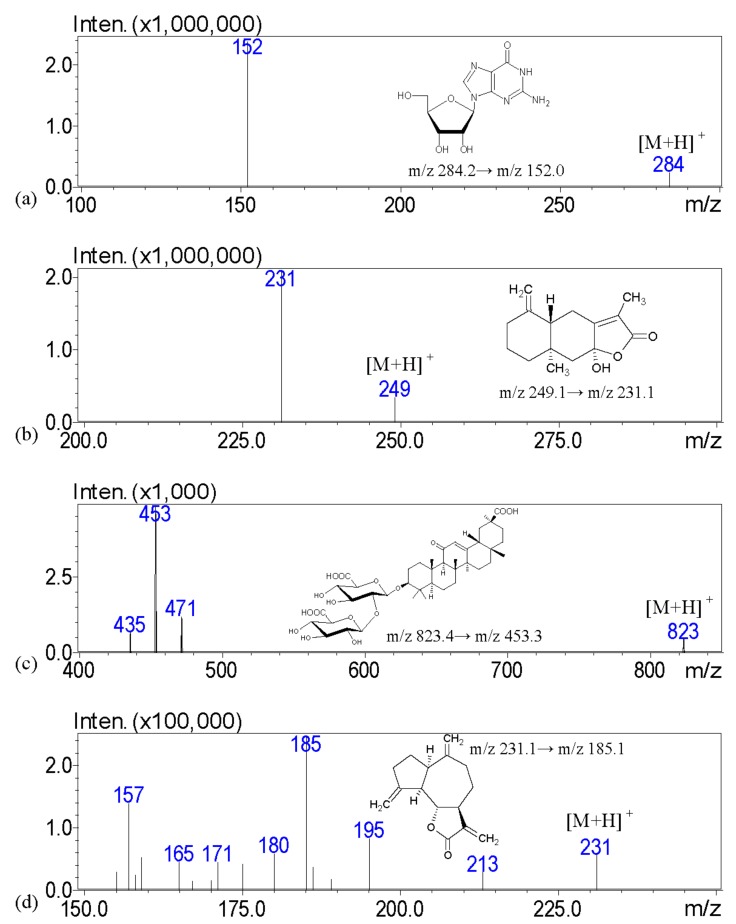

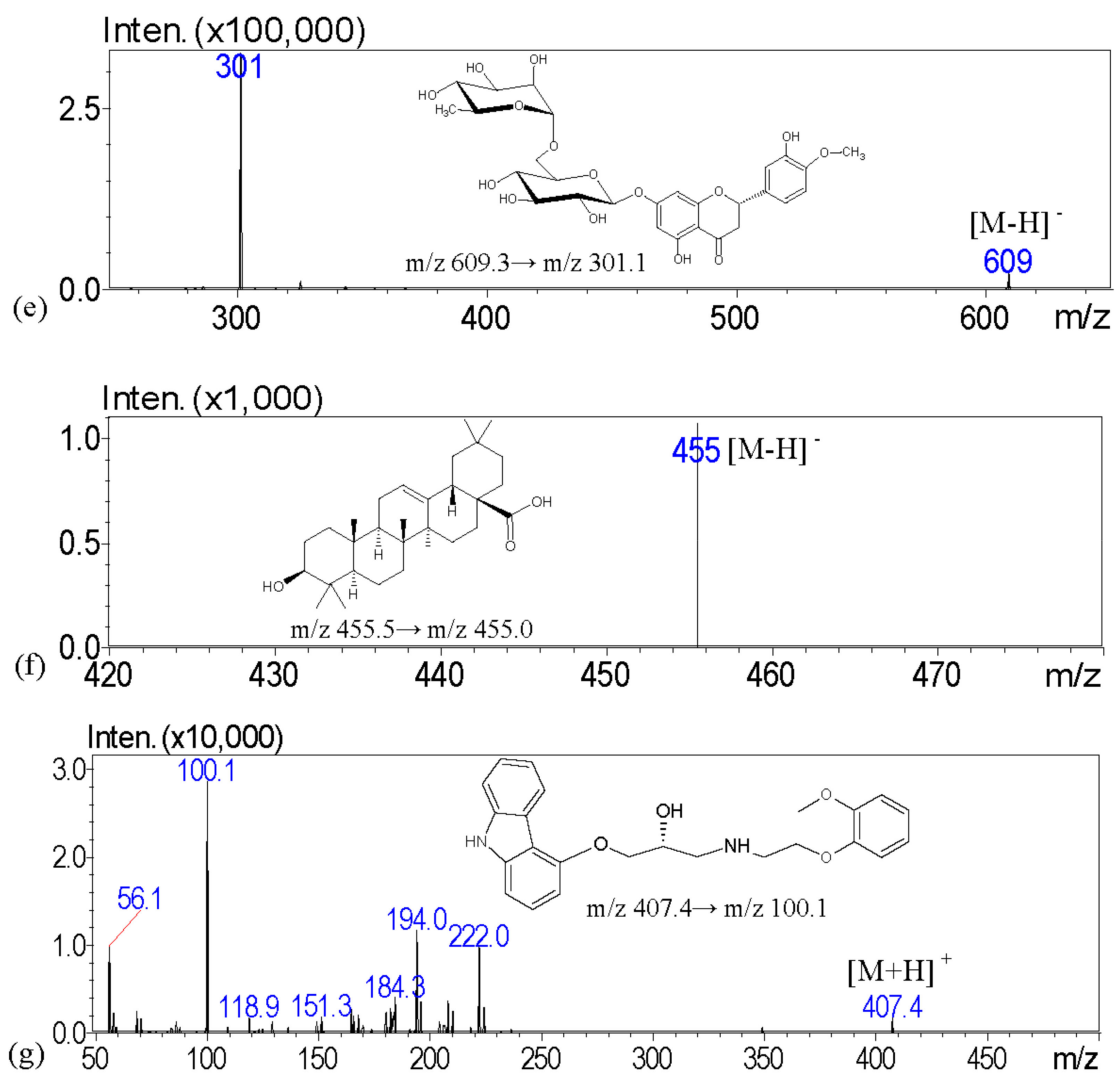
The product ion mass spectra of six marker compounds, including (**a**) guanosine; (**b**) atractylenolide III; (**c**) glycyrrhizic acid; (**d**) dehydrocostus lactone; (**e**) hesperidin; (**f**) oleanolic acid; and (**g**) carvedilol (internal standard).

Identification and determination of the marked ingredients in XSLJZT were performed by LC-MS/MS. The MS spectra of analytes were simultaneously acquired in both ESI (+) and ESI (−) ionization modes. Analytes were quantified in multiple reaction monitoring (MRM) mode targeting the precursor ion (MS 1) to a specific fragment, which is the product ion (MS 2) (Figure 1). The positive ESI mode of [M + H]^+^ was used to yield the precursor ions of guanosine, atractylenolide III, glycyrrhizic acid, and dehydrocostus lactone. The negative ESI mode of [M − H]^−^ was used to produce the precursor ions of hesperidin and oleanolic acid. The internal standards (carvedilol) were analyzed in positive ESI modes. All of the fragment ions were discovered in our study, and the product ions of compounds were selected with good sensitivity. Detailed mass spectrometry results are summarized in Table 1.

**Figure 2:**
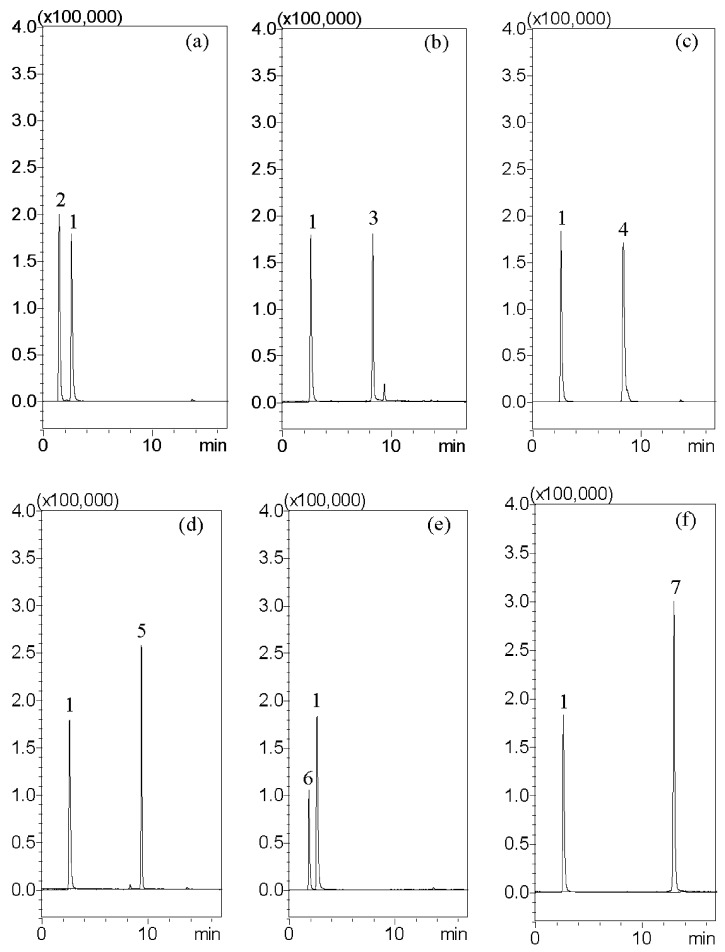
Typical multiple reaction monitoring (MRM) chromatograms of analytes: (**a**) peak two: guanosine (250 ng/mL); (**b**) peak three: atractylenolide III (50 ng/mL); (**c**) peak four: glycyrrhizic acid (1000 ng/mL); (**d**) peak five: dehydrocostus lactone (50 ng/mL); (**e**) peak six: hesperidin (1000 ng/mL); and (**f**) peak seven: oleanolic acid (1000 ng/mL). Peak one: the internal standard is carvedilol (10 ng/mL).

The resolution and peak shapes of the analytes, the elution system, and types of columns were examined while modifying the LC condition. The optimized LC condition included separating analytes with a gradient elution system that consisted of methanol and a 10 mM ammonium acetate solution containing 0.1% formic acid. The Masslynx software differentiates individual transitions from each other; thus, the signal of each analyte would not influence by the other analytes in our study. Besides, all of the analytes were validated with the selectivity, inter-day assay, and intra-day assay for evaluating this developed means. As the following provided chromatographs indicate, they obviously show selectivity and purification. These chromatographic conditions reached the goal of good resolution, good sensitivity, selectivity, and symmetry of the peaks (Figure 2).

In our study, high-performance liquid chromatography coupled with mass spectrometry (HPLC–MS) can be used to establish a high sensitivity means for simultaneously determining various compounds in the herbal samples. HPLC–MS can reach this goal especially for rapid analysis [32]. The selected transition of the analytes was characteristic, and stable fragments were based on the references. Therefore, the resolution of two random peaks in total ion chromatography is not as crucial as in HPLC–UV and HPLC–FLD. As described above, even though massive phenolic acids containing in biosamples, the resolution and elution of each analyte would perform satisfyingly using HPLC–MS. The developed LC–MS methods of XSLJZT in our study were more suitable for analysis because the analysis time was short, and there was clear peak separation, and undisturbed isolation compared with Wang’s study [33]. This optimized method may provide a standard procedure of quality and quantity control for Chinese herbal commercial manufacturers.

### 2.2. Method Validation

Good linearity was achieved for the calibration curve of each analyte. The ranges for the calibration curves for each analyte were as follows: 100–2500 ng/mL for guanosine, 50–1000 ng/mL for atractylenolide III, 50–1000 ng/mL for glycyrrhizic acid, 50–1000 ng/mL for dehydrocostus lactone, 50–1000 ng/mL for hesperidin, and 50–1000 ng/mL for oleanolic acid. The calibration curves and correlation coefficients (*r*^2^) were described in Table 2. For the calibration curves, X represents the concentration of each compound, and y represents the peak area ratio. All of the correlation coefficients (*r*^2^) of the six constituents were greater than 0.995.

The data show that the limits of detection (LOD) of the analytes were 0.5 ng/mL (atractylenolide III), 1 ng/mL (guanosine and dehydrocostus lactone), 5 ng/mL (glycyrrhizic acid and oleanolic acid) and 10 ng/mL (hesperidin), respectively. The lower limits of quantitation (LLOQ) were set at 100 ng/mL for guanosine and 50 ng/mL for atractylenolide III, glycyrrhizic acid, dehydrocostus lactone, hesperidin, and oleanolic acid (Table 2).

The relative standard deviation (RSD) values for the inter-day and intra-day measurements reflect the precision and the percentage of differences between the nominal concentration, and the observed concentrations for inter-day and intra-day measurements reflected the accuracy. The relative standard deviation (RSD) values were found to be within the ranges of 0.69–6.66% for intra-day assays, and 0.47–11.8% for inter-day assays, with accuracy ranges of −13.1–6.43% and −15.3–7.26%, respectively. The results indicated the precision and accuracy values were within ±15%, which were considered acceptable in the experimental concentration range, and the LLOQ values were less than ±20%, which is summarized in Table 3.

### 2.3. Quantitative Determination of the Six Marker Ingredients of XSLJZT products

To investigate the amounts of the marker ingredients in commercially available XSLJZT products from various pharmaceutical manufacturers, optimized and validated techniques were selected for quantitative analysis. The results include the contents of guanosine, atractylenolide III, glycyrrhizic acid, dehydrocostus lactone, hesperidin, and oleanolic acid in six brands, A–F, and a lab-prepared herbal extract, which had ranges from 0.03 to 0.09 mg/g, 0.03 to 0.22 mg/g, 0.30 to 0.85 mg/g, 0.05 to 0.45 mg/g, 4.06 to 11.62 mg/g and 0.02 ± 0.01 mg/g, respectively (Table 4). Oleanolic acid was rarely found in brands A–F, but it was detectable in lab-prepared herbal extract samples. The contents of the marked ingredients in all of the tested samples are shown in Table 4. LC–MS with chemical profiling was provided to rapidly evaluate the chemical consistency among the herbal pharmaceutical products of XSLJZT.

### 2.4. Physical Examination of the Pharmaceutical Additives

#### 2.4.1. Scanning Electron Microscopy (SEM)

**Figure 3:**
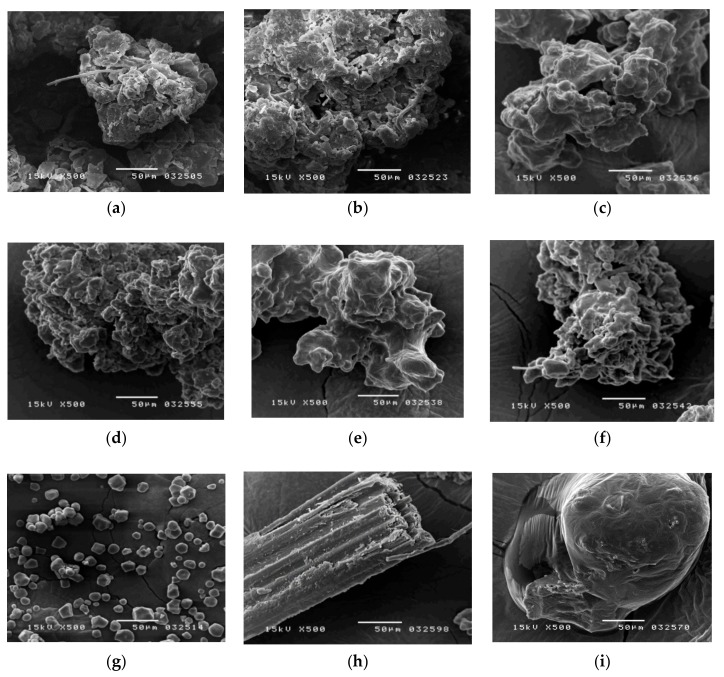
Scanning electron microscope photographs of the (**a**) herbal pharmaceutical product XSLJZT of brand A; (**b**) herbal pharmaceutical product XSLJZT of brand B; (**c**) herbal pharmaceutical product XSLJZT of brand C; (**d**) herbal pharmaceutical product XSLJZT of brand D; (**e**) herbal pharmaceutical product XSLJZT of brand E; (**f**) herbal pharmaceutical product XSLJZT of brand F; (**g**) cornstarch; (**h**) raw herbal powder; and (**i**) raw herbal extraction (×500).

To observe the morphology of the traditional Chinese medicine powder, scanning electron microscopy (SEM) was used. The SEM photographs not only showed the outer appearance of the powder samples (Figure 3a–f), they also showed the characteristics of the particles, such as crude and irregular particle-merged shapes due to gelatinization during the manufacturing process. Compared to the herbal pharmaceutical products of XSLJZT, the shape of cornstarch (Figure 3g) was oval, smooth, and granular. In this investigation, the raw herbal powder showed crushed botanical fibers from plant rhizomes that appeared stripy, stretched, and filiform, as shown in the photograph of raw herbal powder (Figure 3h). These results demonstrated that cornstarch and the raw herbal powder of XSLJZT products could be distinguished clearly by SEM analysis.

#### 2.4.2. Light Microscopy Images of Congo Red and Iodine–KI Stained Samples

**Figure 4:**
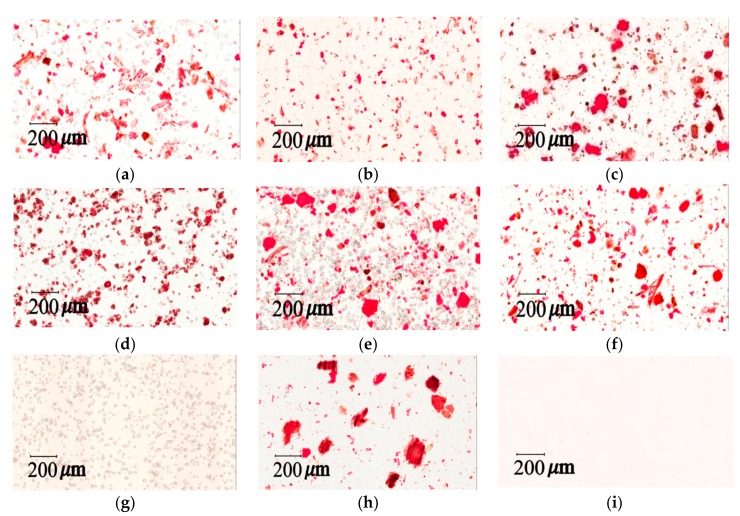
Light microscopy photographs of Congo red staining: (**a**) herbal pharmaceutical product XSLJZT of brand A; (**b**) herbal pharmaceutical product XSLJZT of brand B; (**c**) herbal pharmaceutical product XSLJZT of brand C; (**d**) herbal pharmaceutical product XSLJZT of brand D; (**e**) herbal pharmaceutical product XSLJZT of brand E; (**f**) herbal pharmaceutical product XSLJZT of brand F; (**g**) cornstarch; (**h**) raw herbal powder; and (**i**) raw herbal extraction (×100).

**Figure 5:**
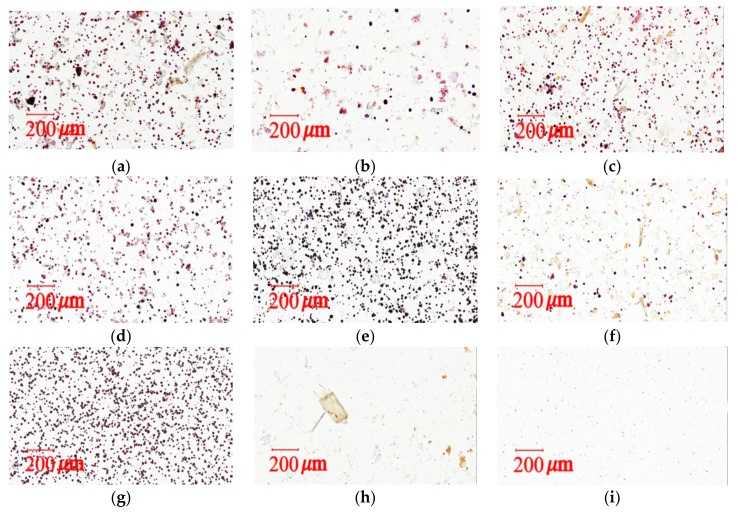
Light microscopy photographs of iodine–KI staining: (**a**) herbal pharmaceutical product XSLJZT of brand A; (**b**) herbal pharmaceutical product XSLJZT of brand B; (**c**) herbal pharmaceutical product XSLJZT of brand C; (**d**) herbal pharmaceutical product XSLJZT of brand D; (**e**) herbal pharmaceutical product XSLJZT of brand E; (**f**) herbal pharmaceutical product XSLJZT of brand F; (**g**) cornstarch; (**h**) raw herbal powder; and (**i**) raw herbal extraction (×100).

Congo red has recently been used to identify cellulose fibril content in phytochemistry [34]. Through noncovalent affinity and synthesizing a red complex, Congo red has a strong interaction with polysaccharides [31]. An Aperio ScanScope slide scanner was used to confirm and identify cellulose fibers through Congo red staining. The herbal pharmaceutical products that contained cellulose fibers were stained red (Figure 4a–f). In contrast, cornstarch (Figure 4g) and raw herbal extracts (Figure 4i) were not dyed red, because there is no fiber in the content. The results imply that samples A to F contained fiber constituents, which suggests the possible use of raw herbal powder or cellulose fiber as additives. The iodine–potassium iodide staining method was applied to evaluate the starch content in the pharmaceutical products. In the presence of iodine–KI, the amylose in starch is responsible for the formation of a deep blue color. The triiodide ion complex slips inside the coil of the starch and creates a strong blue–black color [35]. Our study assessed the starch identification method by light microscopy photographs using iodine–KI reagent staining. The results of iodine–KI staining disclosed an unequal amount of starch content between different brands of XSLJZT (Figure 5a–f). Additionally, the cornstarch solution contained the highest amount of starch (Figure 5g), while no cornstarch was observed in raw herbal powder or raw herbal extract (Figure 5i).

#### 2.4.3. Quantitation of Additives and Starch Granules in Raw Herbal Powder

A series of physical examinations were performed to assess the starch and raw herbal content in the pharmaceutical herbal products. Based on previous analysis [9,31,35], data analysis can be a rapid and reliable indicator of quality control, and it can be used to determine whether raw herbal powders and starch were added to XSLJZT. The photograph of KI-stained cornstarch was used as a positive control to confirm the normal condition of cornstarch for this study. The starch contents of samples A–F were explicitly observed under a microscope using iodine–KI reagent staining, and Aperio Image Scope software was used to calculate the diameter of the particles of cornstarch (12.91 ± 0.09 μm) and large starches from brand B (14.81 ± 0.22 μm) within a fixed area. The results indicate that brands A–F contained cornstarch and raw herbal powders with particle numbers of 36–610 and 42–145, and particle sizes of 13.06–14.81 and 22.87–44.35 μm, respectively (Table 5). As shown in Figure 5, the photographs of brands A, C, D, and E had purple spots, which suggests that these four brands contained abundant amounts of cornstarch. Additionally, the photographs of brands A and E presented in Figure 4 reveal that these six brands generally contained herbal powder. Raw herbal extract is a clear liquid that results after filtration. Thus, no starch granules or raw herbal powders were detected under the microscope.

#### 2.4.4. Solubility and Swelling Experiment

When merchants are concerned about stability and dispersity, adding cornstarch and raw herbal powder are strategies to enhance these qualities in herbal products [35]. The swelling test is used for observing the water absorption ability of starch. Determining the content of starch granules demonstrates the degree of hygroscopic swelling. Differences among starch granules may also result in different patterns of swelling power and solubility. To investigate the physical properties of starch in the pharmaceutical products of XSLJZT, the solubility and swelling tests were conducted at different temperatures using a heated water bath. The results indicated that the solubility ranges of herbal pharmaceutical powders produced by manufacturers A–F were 29.38–34.12% at 55 °C, 32.52–35.91% at 65 °C, 33.55–40.62% at 75 °C, 32.87–43.95% at 85 °C, and 33.91–49.38% at 95 °C (Table 6). Meanwhile, the swelling values of the pharmaceutical products for brands A–F were 1.90–3.50%, 3.00–3.60%, 2.50–6.41%, 4.22–9.23%, and 4.19–13.86%, respectively (Table 7). Importantly, for cornstarch, the solubility was 36.59–67.96%, and the swelling was 5.48–19.64%, which indicates that the swelling increased with temperature. This finding confirms that the swelling of cornstarch is proportional to temperature, which was also reported in a previous study [36]. Therefore, the cornstarch will increase the relative proportion of starch content. In pharmaceutical products A–F, no significant increases in the solubility or swelling were observed with increasing temperature. Remarkably, the expansions of brand A and E were significant at elevated temperatures in direct proportion to the higher starch content. However, the solubility and swelling changes of the six manufactured products only occurred within a limited range. Therefore, we suggest that the solubility and swelling data provide indirect information on the starch content of the pharmaceutical products.

#### 2.4.5. Crude Fiber Content

According to a previous study, crude fiber analysis was considered to be a believable indicator for the determination of the amount of raw herbal powder added to a composition [31]. The evaluation of added raw herbal powder was confirmed using crude fiber analysis. The crude fiber contents for the cornstarch, the whole raw herbal powder, and the raw herbal extract were 0.62 ± 0.31%, 56.09 ± 2.54%, and 3.39 ± 0.52%, respectively. It was difficult to find crude fiber in the cornstarch in this experiment, because cornstarch was reported to contain almost no crude fiber [37]. The raw herbal powders were ground from crushed roots, and they reflect the maximum cellulose fiber substance in the preparations. Since the raw herbal extract was filtered through a 60-mesh sieve, there was little cellulose fiber in the sample. The crude fiber contents of the pharmaceutical products for brands A–F were 24.84 ± 6.65%, 19.10 ± 8.22%, 23.28 ± 4.21%, 36.98 ± 7.51%, 31.23 ± 6.79%, and 25.64 ± 8.45%, respectively. In conclusion, these pharmaceutical herbal products all contain different percentages of raw herbal powder.

## 3. Materials and Methods

### 3.1. Reagents and Materials

Guanosine, atractylenolide III, glycyrrhizic acid, dehydrocostus lactone, hesperidin, oleanolic acid, and carvedilol were purchased from Sigma-Aldrich Chemicals (St. Louis, MO, USA). LC/MS-grade methanol, formic acid, and ammonium acetate were obtained from E. Merck (Darmstadt, Germany). Deionized water (Millipore, Bedford, MA, USA) was used throughout the entire experiment. Crushed XSLJZT herbs were purchased from a Chinese traditional herbal medicine store in Taipei, and prepared at the National Research Institute of Chinese Medicine, Taipei, Taiwan. The herbal pharmaceutical products of XSLJZT were purchased from six different pharmaceutical manufacturers in Taiwan, which included Sun Ten Pharmaceutical Co., Ltd. (Taipei, Taiwan), Kaiser Pharmaceutical Co., Ltd. (Tainan, Taiwan), Chuang Song-Zong Pharmaceutical Co., Ltd. (Kaohsiung, Taiwan), Koda Pharmaceutical Co., Ltd. (Taoyung, Taiwan), Sheng Chang Pharmaceutical Co., Ltd. (Taipei, Taiwan), and Jin-An Pharmaceutical Co., Ltd. (Chiayi, Taiwan). For the results of the analysis, coding was applied for the six manufacturers to preserve commercial confidentiality.

### 3.2. High-Performance Liquid Chromatography–Tandem Mass Spectrometry

HPLC–MS analysis was carried out with a Shimadzu LCMS-8030 triple quadrupole mass spectrometer equipped with an electrospray ionization interface and integrated into the UPLC system (Shimadzu, Kyoto, Japan). The optimization of the instrument settings was as follows: interface voltage, 4.5 kV; desolvation line temperature, 250 °C; heat block temperature, 400 °C; desolvation gas, nitrogen; desolvation gas flow rate, 3 L/min; drying gas, nitrogen; drying gas flow rate, 15 L/min; collision gas, argon; and collision gas pressure, 230 kPa. Multiple reaction monitoring (MRM) mode was used in the MS spectrometer operating parameters. The chromatographic separation was accomplished using a Purospher^®^ STAR RP-18 end-capped column (100 mm × 2.1 mm, two μm, Merck KGaA, Darmstadt, Germany). The column temperature was maintained at 40 °C. The following gradient program was used, along with a mobile phase consisting of 10 mM ammonium acetate in 0.1% formic acid (pH: 3.4) (solvent A) and methanol (solvent B): this initial term for 3 min in an isocratic elution composing with 60% solvent B; 3–8 min: 60–95% B; 8–13.5 min: 95–95% B; 13.5–15 min: 95–60% B; and 15–17 min: 60–60% B; *v*/*v*. The flow rate was 0.2 mL/min, and the injection volume was 10 μL.

### 3.3. Standard Solutions

The stock solutions were formed by dissolving 1 mg of guanosine, atractylenolide III, glycyrrhizic acid, dehydrocostus lactone, hesperidin, and oleanolic acid into 1 mL of 100% (*v*/*v*) methanol to a final concentration of 1.0 mg/mL, respectively. All of the stock solutions were stocked at −20 °C before use. To prepare a series of working standard solutions, the stock solution was diluted with 50% methanol to obtain the following concentrations: 50, 100, 250, 500, and 1000 ng/mL. Guanosine was assayed at concentrations of 100, 250, 500, 1000, and 2500 ng/mL. Working solutions for quality control (QC) samples with five level concentrations of 50, 100, 250, 500, and 1000 or 2500 ng/mL were prepared in the same manner. All of the solutions were kept at 4 °C and brought to room temperature before analysis.

### 3.4. Sample Preparation for Extracts of Herbal Pharmaceutical Powders

The classic TCM prescriptions written by well-known ancient and modern physicians originally described Xiang-Sha-Liu-Jun-Zi-Tang (XSLJZT), which is used after boiling herbs in water. Therefore, we followed the ancient recipe of boiling herbs to extract raw herbs practicing in the lab extraction. However, the herbal ingredients may not be exactly the same extraction in the lab, due to the complexities of herbal plants.

*Radix Aucklandiae* (7 g), *Fructus Amomi* (3 g), *Radix ginseng* (10 g), *Atractylodes Rhizoma* (20 g), *Poria cocos* (Schw.) *Wolf* (20 g), *Glycyrrhizae radix* (7 g), *Pericarpium Citri Reticulatae* (3 g), *Rhizoma Pinelliae* (12 g), and *Ziziphus jujuba* (four pieces) were extracted with 700 mL of boiling water for 60 min. The decoction was filtered, and the solution was freeze-dried under vacuum and dissolved in 100% MeOH for analysis. Each specimen was prepared using 0.1 g of Chinese pharmaceutical herbal powder or dried XSLJZT decoction powder steeped in 25 mL of methanol (4 mg/mL), which was then extracted in an ultrasonic oscillator for 20 min at room temperature and centrifuged at 13,000× *g* rpm for 10 min at 4 °C. The supernatant was collected through a 0.22 μm syringe filter, diluted to a suitable concentration, and infused into the HPLC–MS for analysis. However, Chinese medicine powder is produced with granulating via boiled soups, so the practicing method in this study using alcohol extraction is reasonable. Consequently, herbal preparation (A–F) and the lab extraction samples adopted the same extraction method.

### 3.5. Quantitative Determination of Active Compounds

Quantitative determination was completed by using the most intense ion detected. The interpolation method of the calibration curve was adopted to determine the relative concentration of active compounds in each sample, and each batch of samples correlated with the same calibration curve. Using back calculation, the content of active compounds in Xiang-Sha-Liu-Jun-Zi-Tang was calculated using the following formula. The content of active compounds in XSLJZT (mg/g) = [determined concentration (ng/mL)/concentration of sample (4 mg/mL)] × dilution ratio.

### 3.6. Method Validation

The HPLC–MS method was validated based on the recommendations published by the Food and Drug Administration (FDA) Guidance for Industry, Bioanalytical Method Validation [38]. The correlation coefficients (*r*^2^) of all of the calibration curves had good linearity, with values greater than 0.995. The limit of detection (LOD) was defined as the concentration that yields a signal-to-noise ratio of three, and the lower limit of quantification (LLOQ) was accepted as the lowest concentration of the linear regression. The intra-day and inter-day variations were determined by analyzing six replicates on the same day and on six successive days, respectively. The precision of the observed concentrations was calculated by relative standard deviation (RSD) (%) = (standard deviation (SD)/Cobs) × 100%. The accuracy was achieved by calculating the bias (%) = [(Cobs − Cnom)/Cnom] × 100%. Cobs represented the mean value of the observed concentrations, and Cnom represented the nominal concentration. The acceptable values of accuracy and precision were within ±15%, and the values of LLOQ were less than ±20%.

### 3.7. Physical Examination of Additives for Raw Herbal Powder

#### 3.7.1. Estimation of Additives for Raw Herbal Powder

To observe the morphology of the samples, a scanning electron microscope (SEM) was used. The herbal pharmaceutical products of XSLJZT were purchased from six different pharmaceutical manufacturers in Taiwan. The crushed herbs of XSLJZT were obtained from a Chinese traditional herbal medicine shop in Taipei and formed a raw herbal powder in the National Research Institute of Chinese Medicine, Taipei, Taiwan. These herbs were filtered through a 60-mesh sieve after they were ground by the hammer mill (Hung Chuan RT-04, Taipei, Taiwan). Food-grade cornstarch (Sun Right Co., Ltd., New Taipei, Taiwan) was filtered through a 60-mesh sieve. For sample preparation, the herbal pharmaceutical powder was dried at 45 °C for 24 h in an oven (DO45, DENYNG Instruments Co., Ltd., New Taipei, Taiwan). Then the powder was fixed with double-sided adhesive tape on an aluminum stand, and coated with gold by a gold sputter package for 90 s in a high-vacuum evaporator (Ion Sputter JFC-1200, Jeol Ltd., Tokyo, Japan). A scanning electron microscope (JEOL JSM-5300, Jeol Ltd., Tokyo, Japan) was used to analyze the samples. The raw herbal powder, raw herbal extraction, and cornstarch were investigated in the same manner.

#### 3.7.2. Light Microscopy Photographs of Congo Red and Iodine–KI Stained Samples

The iodine test for starch is used to determine the presence of starch in materials [9,31,35,39]. The affinity of Congo red is high for fibers. It has been widely used to test media additives, biological staining, plant mucin, and cellulose [40]. Congo red and Iodine–KI staining tests can provide qualitative and quantitative measurements for cellulose fibers and cornstarch in herbal pharmaceutical products. Light microscopy images were taken with a light microscope (Aperio Scanscope CS system, Aperio, CA, USA). Samples were prepared in a mixed solution (glycerol/20% ethanol (1:1)) and placed on microslides by adding 1–2 drops of the solution. The preparation was then covered with a coverslip for viewing and stained with 0.1% Congo red or 2% iodine–KI solution.

The measurement of total raw herbal powder and cornstarch in six different herbal pharmaceutical XSLJZT products was determined by counting the number of staining spots for raw herbal powder and cornstarch. Acceptance criteria: The cornstarch is generally represented by rounded or spheroidal granules with diameters ranging from 10 μm–15 μm, and the raw herbal powder contained longed and fragmented cellulose fibers with diameters ranging from 20 μm–120 μm that could be clearly observed under the microscope after 10% iodine–KI and 0.1% Congo red staining.

#### 3.7.3. Solubility and Swelling Test

The water solubility and swelling power index experiments of the herbal pharmaceutical powders were modified from previous reports [9,31,35]. The respective brands of XSLJZT powder samples (0.45 g) were added to 30 mL of distilled water (30 g, 1.5%, *w*/*w*). These XSLJZT sample solutions were vortexed and heated to 55 °C, 65 °C, 75 °C, 85 °C, and 95 °C sequentially for one hour each in a circulating water bath (BH-230D, YIN DER Instruments Co., Ltd., New Taipei, Taiwan). After heating, the samples were cooled to room temperature and centrifuged at 13,000× *g* at 4 °C for 20 min to separate the supernatant and sediment precipitations. The supernatant was collected and placed in a drying oven (DO45, DENGYNG Instruments Co., Ltd., New Taipei, Taiwan) at 100 °C; and the weight of the residue and the residue precipitate in the centrifuge tube were represented by W1 and W2, respectively. The solubility was calculated with the following formula: solubility = (W1/pharmaceutical herbal powder weight) × 100%. Swelling power = W2/[powder weight × (1 − Solubility/100)].

#### 3.7.4. Crude Fiber Analysis

The crude fiber content of the XSLJZT powder samples were determined using a method from the Crude Fiber in Flours, Feeds, and Feedstuffs Method of the American Association of Cereal Chemists (AACC) [41]. The XSLJZT herbal powder sample (2 g) was boiled for 30 min in 200 mL of 1.25% H_2_SO_4_, washed with hot distilled water, and filtered with a suction apparatus. Then, the sample was transferred to a boiled 1.25% NaOH solution and treated in the same manner. The residue was desiccated at 100 °C for 24 h in an oven to achieve constant weight and burned at 550–600 °C for 5–6 h in a muffle furnace (DF202, DENGYNG Instruments Co., Ltd., New Taipei City, Taiwan) until gray ash was collected. The crude fiber (%) was calculated by the formula: crude fiber (%) = (constant weight of residue − the weight of ash) × 100/weight of the sample.

### 3.8. Statistical Analysis

The microscopy photograph analysis was completed with an Aperio ScanScope CS (Aperio Technologies, Vista, CA, USA), and the number of particles was calculated by the software Aperio ImageScope (version 10.0). Statistical analyses were performed with SPSS Version 18.0 (SPSS, Chicago, IL, USA) and SigmaPlot 10.0 software. Data are expressed as the mean ± standard deviation. Analysis of variance was conducted by Student’s *t* test or a one-way ANOVA comparison adjustment, and statistically significant differences were defined as 𝑝 < 0.05.

## 4. Conclusions

In this study, a rapid, selective, and validated HPLC–MS method was developed to simultaneously determine the guanosine, atractylenolide III, glycyrrhizic acid, dehydrocostus lactone, hesperidin, and oleanolic acid concentrations in various preparations of Xiang-Sha-Liu-Jun-Zi-Tang. This method has been an effective and specific tool for the identification of complex constituents in herbal medicine. Several chemical and physical methods were established to evaluate the quality of the herbal products. In addition, with Aperio ImageScope software, the quantification of cornstarch and cellulose fiber content can be accomplished by calculating the particle number and size, and this approach can be used for the quality control of commercial herbal powder products. The methods developed in this study may provide a standard procedure for quality and quantity control for Chinese herbal commercial manufacturers.

## Figures and Tables

**Table 1:** The high-performance liquid chromatography–tandem mass spectrometry (HPLC-MS) conditions for identification of the six constituents and one internal standard.

Constituents	Molecular Weight	RT (min)	Ion Mode	CE	Precursor Ion (amu)	Product Ion (amu)
Guanosine	283.24	1.50	+	−15	284.2 [M + H]^+^	152.0
Atractylenolide III	248.32	6.35	+	−9	249.1 [M + H]^+^	231.1
Glycyrrhizic acid	822.94	8.42	+	−30	823.4 [M + H]^+^	453.3
Dehydrocostus lactone	230.3	9.42	+	−11	231.1 [M + H]^+^	185.1
Hesperidin	610.56	1.87	−	25	609.3 [M − H]^−^	301.1
Oleanolic acid	456.7	13.0	−	46	455.5 [M − H]^−^	455.0
Carvedilol (IS)	406.4	2.36	+	−35	407.4 [M + H]^+^	100.1

RT: retention time (unit: minute); CE: collision energy (unit: electron volt); IS: internal standard.

**Table 2:** Linear ranges, calibration curves, correlation coefficients (*r*^2^), and detection limits of six constituents using HPLC–MS.

Constituents	Linear Range (ng/mL)	Calibration Curve	*r*^2^	LLOQ (ng/mL)	LOD (ng/mL)
Guanosine	100–2500	*y* = 0.0093*x* − 0.1005	0.9998	100	1
Atractylenolide III	50–1000	*y* = 0.0170*x* + 0.0566	0.9990	50	0.5
Glycyrrhizic Acid	50–1000	*y* = 0.0010*x* − 0.0024	0.9997	50	5
Dehydrocostus lactone	50–1000	*y* = 0.0191*x* + 0.2153	0.9998	50	1
Hesperidin	50–1000	*y* = 0.0005*x* + 0.0012	0.9997	50	10
Oleanolic acid	50–1000	*y* = 0.0047*x* − 0.0694	0.9996	50	5

LLOQ: lower limit of quantification; LOD: limit of detection was determined at a signal-to-noise ratio (*S*/*N*) of 3.

**Table 3:** Intra-day and inter-day precision and accuracy for the six active components of Xiang-Sha-Liu-Jun-Zi-Tang (XSLJZT). RSD: relative standard deviation.

Nominal Conc. (ng/mL)	Intra-Day			Inter-Day		
	Observed Conc. (ng/mL)	RSD (%)	Bias (%)	Observed Conc. (ng/mL)	RSD (%)	Bias (%)
Guanosine						
100	93.86 ± 1.95	2.08	−6.14	95.15 ± 11.2	11.8	−4.85
250	241.5 ± 16.1	6.66	−3.38	239.4 ± 17.0	7.08	−4.23
500	494.2 ± 17.0	3.44	−1.16	521.3 ± 19.6	3.76	4.26
1000	1024 ± 26.9	2.63	2.40	999.1 ± 14.5	1.45	−0.09
2500	2483 ± 24.0	0.97	−0.69	2491 ± 16.9	0.68	−0.37
Atractylenolide III						
50	49.20 ± 2.86	5.81	−1.60	49.15 ± 2.27	4.62	−1.70
100	97.27 ± 2.42	2.49	−2.73	98.65 ± 4.26	4.32	−1.35
250	255.4 ± 4.77	1.87	2.17	256.5 ± 8.28	3.23	2.60
500	504.0 ± 9.92	1.97	0.80	505.1 ± 6.22	1.23	1.21
1000	1004 ± 12.6	1.26	0.38	999.1 ± 8.97	0.90	−0.08
Glycyrrhizic Acid						
50	47.55 ± 2.87	6.03	−4.90	50.87 ± 3.99	7.84	1.75
100	102.5 ± 4.02	3.93	2.46	104.9 ± 5.51	5.25	4.87
250	255.0 ± 6.67	2.62	2.00	259.6 ± 6.72	2.59	3.85
500	502.3 ± 15.7	3.12	0.46	502.3 ± 29.92	6.00	−0.29
1000	1008 ± 22.5	2.23	0.84	1022 ± 29.50	2.89	2.15
Dehydrocostus lactone						
50	43.47 ± 1.90	4.36	−13.1	42.37 ± 1.76	4.15	−15.3
100	100.3 ± 3.31	3.29	0.34	100.5 ± 3.97	3.95	0.48
250	260.2 ± 7.22	2.77	4.08	253.9 ± 4.64	1.83	1.55
500	501.2 ± 10.2	2.04	0.24	508.7 ± 12.7	2.50	1.74
1000	1002 ± 9.75	0.97	0.22	995.9 ± 4.70	0.47	−0.41
Hesperidin						
50	49.93 ± 1.03	2.06	−0.13	47.77 ± 3.84	8.04	−4.46
100	104.5 ± 3.39	3.24	4.47	103.0 ± 5.11	4.96	3.04
250	257.0 ± 4.66	1.81	2.81	256.1 ± 9.05	3.53	2.45
500	474.9 ± 6.22	1.31	−5.02	490.4 ± 8.06	1.64	−1.93
1000	1003 ± 6.96	0.69	0.33	999.5 ± 30.6	3.06	−0.05
Oleanolic acid						
50	47.47 ± 1.98	4.16	−5.05	44.14 ± 1.02	2.32	−11.7
100	106.4 ± 2.67	2.51	6.43	107.3 ± 2.13	1.98	7.26
250	240.4 ± 7.14	2.97	−3.83	251.3 ± 3.99	1.59	0.53
500	498.5 ± 17.5	3.52	−0.30	492.2 ± 7.03	1.43	−1.56
1000	992.2 ± 21.64	2.18	−0.78	998.7 ± 8.55	0.86	−0.13

Data are expressed as the means ± SD. Precision (%RSD) = (S.D./C_obs_) × 100. Accuracy (%Bias) = [(C_obs_ − C_nom_)/C_nom_] × 100.

**Table 4:** The contents of guanosine, atractylenolide III, glycyrrhizic acid, hesperidin, dehydrocostus lactone, and oleanolic acid in a lab-prepared herbal extract, and six brands (A–F), of XSLJZT herbal pharmaceutical products.

Components (ng/g)	Lab Extraction	A	B	C	D	E	F
Guanosine	0.09 ± 0.03	0.04 ± 0.01	0.03 ± 0.01	0.09 ± 0.01	0.03 ± 0.01	0.09 ± 0.01	0.08 ± 0.01
Atractylenolide III	0.11 ± 0.02	0.07 ± 0.01	0.15 ± 0.02	0.22 ± 0.01	0.03 ± 0.01	0.16 ± 0.01	0.14 ± 0.01
Glycyrrhizic acid	0.81 ± 0.28	0.34 ± 0.07	0.30 ± 0.05	0.84 ± 0.04	0.30 ± 0.03	0.85 ± 0.08	0.71 ± 0.10
Dehydrocostus lactone	0.37 ± 0.23	0.34 ± 0.09	0.21 ± 0.01	0.45 ± 0.11	0.05 ± 0.02	0.13 ± 0.02	0.24 ± 0.26
Hesperidin	4.06 ± 1.99	6.56 ± 1.37	7.60 ± 1.73	8.64 ± 1.36	5.25 ± 0.86	10.35 ± 1.44	11.62 ± 1.83
Oleanolic acid	0.02 ± 0.01	ND	ND	ND	ND	ND	ND

Each value is expressed as the mean ± SD (*n* = 3), and the unit is mg/g. ND: no detection. The sample brands A–F represent XSLJZT herbal pharmaceutical products that were purchased from six different manufacturers.

**Table 5:** Congo red and Iodine–KI staining analysis of total raw herbal powder numbers, total cornstarch numbers, and average size.

Congo Red Staining			Iodine–KI Staining	
Brand	Total Raw Herbal Powder (Number)	Average Size (μm)	Total Cornstarch (Number)	Average Size (μm)
A	84.00 ± 11.53	34.45 ± 1.18	338.0 ± 12.53	13.75 ± 0.65
B	42.67 ± 14.19	22.87 ± 6.56	36.00 ± 3.61	14.81 ± 0.22
C	79.67 ± 80.50	44.35 ± 1.12	279.6 ± 5.86	13.06 ± 0.61
D	145.0 ± 15.13	31.49 ± 0.88	205.3 ± 12.86	13.73 ± 0.35
E	114.6 ± 09.71	37.44 ± 0.46	610.3 ± 2.52	13.19 ± 0.07
F	86.00 ± 13.11	35.68 ± 0.58	57.33 ± 8.74	13.47 ± 0.06
G	00.00 ± 0.000	00.00 ± 0.000	979.3 ± 14.98	12.91 ± 0.09
H	31.33 ± 10.02	52.16 ± 11.28	00.00 ± 0.000	00.00 ± 0.000
I	00.00 ± 0.000	00.00 ± 0.000	00.00 ± 0.000	00.00 ± 0.000

Each value is expressed as the mean ± SD (*n* = 3). The sample brands A–F represent the XSLJZT herbal pharmaceutical products purchased from six different manufacturers; G: cornstarch; H: raw herbal powder; I: raw herbal extraction.

**Table 6:** Solubility analysis of herbal pharmaceutical products at different temperatures.

Solubility (%)					
Brand	55 °C	65 °C	75 °C	85 °C	95 °C
A	33.69 ± 2.08	32.62 ± 2.16	40.62 ± 9.29	39.14 ± 2.61	41.98 ± 1.38
B	31.21 ± 0.35	32.56 ± 5.54	33.55 ± 5.46	32.87 ± 0.25	33.91 ± 1.28
C	33.22 ± 3.04	35.91 ± 5.66	35.69 ± 5.06	36.99 ± 4.17	40.13 ± 7.26
D	29.38 ± 5.14	33.05 ± 1.67	34.15 ± 2.05	36.34 ± 1.34	38.02 ± 1.61
E	34.12 ± 4.86	33.92 ± 3.04	39.10 ± 2.93	43.95 ± 2.81	49.38 ± 0.70
F	32.94 ± 0.43	32.52 ± 2.18	34.30 ± 2.37	35.24 ± 2.19	36.52 ± 1.30
G	36.59 ± 1.53	37.98 ± 1.64	44.53 ± 3.28	52.37 ± 5.67	67.96 ± 4.65
H	29.06 ± 9.48	32.86 ± 0.96	32.13 ± 2.40	31.72 ± 3.49	32.05 ± 1.47
I	35.50 ± 5.02	32.48 ± 1.11	39.48 ± 6.15	37.53 ± 4.34	41.59 ± 8.16

Each value is expressed as the mean ± SD (*n* = 3). Solubility = (the weight of residue/pharmaceutical herbal powder weight) × 100%. The sample brands A–F represent the XSLJZT herbal pharmaceutical products purchased from six different manufacturers; G: cornstarch; H: raw herbal powder; I: raw herbal extraction.

**Table 7:** Swelling analysis of herbal pharmaceutical products at different temperatures.

Swelling (%)					
Brand	55 °C	65 °C	75 °C	85 °C	95 °C
A	2.01 ± 0.16	3.46 ± 0.29	5.17 ± 0.64	8.06 ± 0.60	10.6 ± 0.96
B	1.90 ± 0.22	3.45 ± 0.25	2.50 ± 0.87	4.22 ± 0.36	4.19 ± 0.45
C	2.80 ± 0.61	3.45 ± 0.39	6.16 ± 0.66	6.71 ± 0.32	8.55 ± 0.55
D	3.50 ± 0.54	3.60 ± 0.28	5.07 ± 0.17	6.10 ± 0.29	7.73 ± 0.40
E	2.81 ± 0.29	3.25 ± 0.19	6.41 ± 0.45	9.23 ± 0.26	13.9 ± 1.37
F	2.19 ± 0.57	3.00 ± 0.27	3.35 ± 0.51	4.69 ± 0.14	5.56 ± 0.44
G	5.48 ± 0.46	8.55 ± 0.26	12.2 ± 0.36	14.2 ± 1.04	19.6 ± 1.97
H	0.50 ± 0.13	0.69 ± 0.23	0.79 ± 0.21	0.84 ± 0.09	1.13 ± 0.07
I	1.10 ± 0.24	1.36 ± 0.23	1.65 ± 0.21	1.82 ± 0.32	2.04 ± 0.17

Each value is expressed as the mean ± SD (*n* = 3). Swelling power = the residue precipitate in the centrifuge tube/[powder weight × (1 − Solubility/100)]. The sample brands A–F represent the XSLJZT herbal pharmaceutical products purchased from six different manufacturers; G: cornstarch; H: raw herbal powder; I: raw herbal extraction.
